# Determination of Aflatoxin M1 Levels in Produced Pasteurized Milk in Ahvaz City by Using HPLC

**Published:** 2012-05-28

**Authors:** Abdolazim Behfar, Zahra Nazari Khorasgani, Ziyaaddin Alemzadeh, Mehdi Goudarzi, Rezvan Ebrahimi, Najmedin Tarhani

**Affiliations:** 1 Department of Food Science and Medical Hydrology, Pharmacy School, Ahvaz Jundishapur University of Medical Sciences, Ahvaz, IR Iran; 2 Department of Pharmacology and Toxicology, Pharmacy School, Ahvaz Jundishapur University of Medical Sciences, Ahvaz, IR Iran

**Keywords:** Aflatoxins, Aflatoxin M_1_, Milk, Chromatography, High-Performance, Liquid Chromatography

## Abstract

**Background:**

Aflatoxins are one of the most potent toxic substances that occur naturally. Nowadays extensive attention has been taken to their existence in food and environment, as there is the possibility of harm to humans following chronic exposure to extremely low levels via food chain. Aflatoxin M1 (AFM_1_) is a hepatic carcinogenic metabolite found in the milk of lactating animals fed with contaminated feed contaminated by aflatoxin B1 (AFB_1_).

**Objectives:**

This study aimed to determine the levels of AFM1 in produced pasteurized milk in the Ahvaz of city.

**Materials and Methods:**

For this purpose, 100 samples of pasteurized milk from the Jamus Factory were analyzed the to determine AFM_1_ content by using an immunoaffinity column for clean-up and high-performance liquid chromatography (HPLC) with a C18 column, a fluorescence detector (excitation 365 nm, emission 435 nm) and a mobile phase of acetonitrile–water (25:75, v/v) at a flow rate of 1 mL/min.

**Results:**

AFM_1_ was detected in all 100 samples of pasteurized milk at concentrations ranging from 0.45 to 9.760 ng/L.

**Conclusions:**

The mean concentration of AFM1 in the the pasteurized milk samples was 2.7 ng/L, which was below the 50 ng/L, accepted as level of for milk in Iran.

## 1. Background

Aflatoxins are produced by the Aspergillus species under suitable conditions. They are found in a wide variety of products and commodities, including cereals, peanuts, walnuts, and dried fruits (1-8). Five billion people in developing countries all over the world are at risk of chronic exposure to aflatoxins through contaminated foods (9). One of the metabolites of AFB_1_ by cytochrome P_450_ enzyme system in the liver is 4-hydroxy AFB_1_, (AFM_1_) which is excreted into milk when lactating animals are given feed known to contain aflatoxins (3, 10). The amount of AFM_1_ excreted is directly related to the level of AFB_1_ in the feed. Milk and milk products are good sources of many nutrients such as proteins, calcium, vitamins, and essential fatty acids. On the other hand, contamination of milk with AFM_1_ is considered as a potential risk for human health (11-13). AFM_1_ was classified by the International Agency for Research on Cancer (IRAC) as a group 2B agent (possibly carcinogenic to humans). It has been experimentally shown to confer high hepatotoxic and mutagenic risk. AFM_1_ is relatively stable during pasteurization, sterilization, preparation, and storage of dairy products (13). There is very little data in the literature on AFM_1_ levels in the milk produced in Ahvaz, the capital city of Khouzestan province, Iran. Therefore, it is difficult to estimate the daily intake of AFM_1_ from milk or other dietary sources, thus there is a need to detect and quantify AFM_1_ in milk. Various methods to determine AFM_1_ have been developed, including radioimmunoassay, enzyme-linked immunoassay, and high-performance liquid chromatography (HPLC).

## 2. Objectives

This study was carried out to evaluate AFM_1_ levels in pasteurized milk produced in Ahvaz city by using HPLC.

## 3. Materials and Methods

### 3.1. Chemicals, Reagents, and Materials

AFM_1_ standard was obtained from Sigma Chemical Co. in Iran. Aflatest immunoaffinity columns were purchased from VICAM Co. USA. Acetonitrile HPLC grade was purchased from Merck Co. The stock solution of AFM_1_ was prepared in acetonitrile at a concentration of 0.5 µg/ml and was kept at −20º C. Working standard solutions were prepared by of stock standard solution diluting acetonitrile stock solution at concentrations ranging from 0.05 to 100 ng/ml.

### 3.2. Samples

In this study, 100 composite milk samples, each comprising 5 packs of pasteurized milk, were taken on site at the Jamus Factory from February 2009 to June 2009, and transferred to the Toxicology Lab of the Department of Toxicology and Pharmacology, Pharmacy School of Ahvaz Jundishapur University of Medical Sciences. All samples were stored at −20◦C until analyzed.

### 3.3. Apparatus

The Shimadzu 10ADvp HPLC system (Japan) was equipped with a Shimadzu RF-10AXL fluorescence detector. Shimadzu LC-10 ADvp pump u, isocratic mode, Shimadzu DGU-14A Degasser, Shimadzu SCL-10Avp System Controller, Shimadzu FCL- 10ALvp flow controller, LC solution software. The column (4.6 × 150 mm), which was packed with particles of silica modified with octadecylsilyl groups (5 μm in diameter), was purchased from Capital Co., England.

### 3.4. Clean-up by Immunoaffinity Column Chromatography

Each sample was warmed at 37◦C and centrifuged at 2000×g. The fat layer was removed completely and milk was passed through a paper filter. Then, a 50 ml portion of this prepared sample was taken into a syringe barrel attached to an Aflatest column and passed at the flow rate of 2–3 ml min^−1^. The column was washed with 20 ml of water and discarded. The sorbent bed was dried and the AFM_1_ in the samples was eluted with 4 ml acetonitrile. The solution was evaporated under nitrogen gas and the residue was dissolved in 1 ml of mobile phase.

### 3.5. Quantitative Analysis

The above solution (200 µl) was injected into the HPLC. Excitation and emission wavelengths were 365 nm and 435 nm, respectively. Acetonitrile–water (25:75 v/v) was used as the mobile phase at the flow rate of 1 ml/min. AFM_1_ peak in the chromatogram was identified by comparing its retention time with that of the analyzed AFM_1_ standard under the same conditions. The peak was quantified from the area under the curve of sample chromatogram by using the equation of calibration curve (y = .94481x + 875.9, R^2^= 0.9999). Calibration curve drawn at concentrations of 0.05, 0.1, 0.5, 1, 5, and 10 ng/ml of AFM_1_.

The limits of detection and quantitation were 15.5 and 50 ng/L, respectively. Recovery was performed by the standard addition method. To do so, 18 portions (1 ml each) of 0.1, 0.5, and 1 ng/ml of standard solutions (6 repeats for each level) were transferred into 50 ml volumetric flasks and evaporated under nitrogen gas. The residues in the volumetric flasks were diluted to the mark by adding the required amount of one of the milk samples whose content of AFM_1_ was being analyzed. Then, the procedures above were followed. The results are summarized in Table 1. All recoveries were more than 94%, indicating good accuracy. Intra-day and inter-day precision is shown in Table 2. All measurements were repeated 6 times. The %RSDs of intra-day and inter–day analyses were in the range of 0.334–11.224 and 1.332–11.568, respectively. These data indicate that the method has acceptable precision.

**Table 1:** Recoveries for AFM_1_ From Spiking Into the one of the Milk Samples (n=6)

Sample type	Spiking levels, ppb	Measurable levels, ppb	Recovery, %
Milk	0.1	0.094	94
	0.5	0.48	97
	1	0.96	98

**Table 2:** Intra-day and inter-day Precision of Method (n=6)

AFM_1_ concentration, ng/ml	Intra-day, Mean ± SD (μ V*s)	Precision, RSD, %	Inter-day, Mean ± SD (μ V*s)	Precision, RSD, %
0.05	5158.271±578.073	11.224	5005.05±578.973	11.568
0.1	10479.229±442.672	4.224	10559.90±442.672	4.192
0.5	48398.475±887.005	1.833	48769.78±887.005	1.819
1	99154.367 ±1930.621	1.947	97011.275±1930.621	1.990
5	466528.600±4320.988	0.926	324436.4±4320.988	1.332
10	948743.350±3169.465	0.334	939349.233±16424.708	1.749

## 4. Results

**Figure 1:**
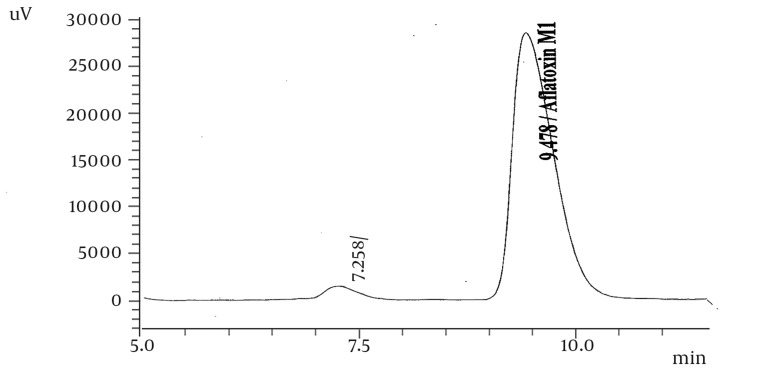
HPLC Chromatogram of 100 ng/ml AFM1 Standard Solution

**Figure 2:**
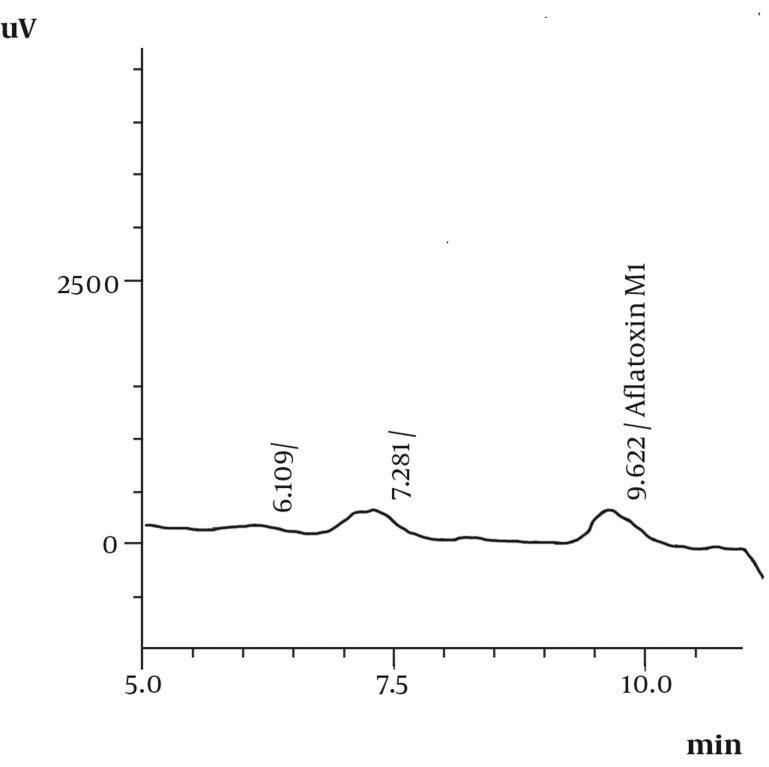
HPLC-FD Chromatogram of Milk Containing Aflatoxin M1.

The average recoveries and relative standard deviation of the analytical method applied for AFM_1_ in milk were investigated. The results are shown in Tables 1 and 2. The highest and lowest concentrations of AFM_1_ were 9.76 and 0.45 ng/L respectively (Table 3). The mean of AFM_1_ concentration in samples was 2.7 ng/L (Table 3). Retention time under this condition was 9.478 ± 0.236min (Figures 1 and 2).

**Table 3:** Descriptive Statistics of Data of Investigated Milk Samples (ng/L)

Type sample	N	Minimum	Maximum	Mean	Std. Deviation	Std. Error of Mean
milk	100	0.45	9.76	2.7	1.878256	0.419991

## 5. Discussion

Since milk and dairy products are an important source of nutrition in the human diet, the presence of AFM_1_ in milk and milk products has been investigated worldwide. In 1996, Galvano, F *et al*. examined for the presence of AFM_1_ in 161 samples of milk, 92 samples of dry milk for infant formula, and 120 samples of yogurt obtained from supermarkets and drug stores in 4 large Italian cities by using immunoaffinity column extraction and HPLC. AFM_1_ was detected in 125 (78%) of milk samples (ranging from <0.001 µg/L to 0.0235 µg/L; mean level 0.00628 µg/L), 49 (53%) of dry milk samples (ranging from <0.001 µg/L to 0.0796 µg/kg; mean level 0.0322 µg/kg), and 73 (61%) of yogurt samples (ranging from <0.001 µg/kg to 0.0321 µg/kg; mean level 0.00906 µg/kg).

Only 4 samples of dry milk were over the legal limit established by the European Community (EC) in 1999 (14). In October–July 2000, Bognanno, M. *et al*. analyzed 240 samples of dairy ewes’ milk from farms in Enna (Sicily, Italy) for AFM_1_ by using HPLC equipped. with a fluorescence detector. The limit of detection was 0.250 µg/L for AFM_1_. All positive milk samples for AFM_1_ were confirmed by LC-MS. AFM_1_ was detected in 81% of milk samples, ranging from 0.002 to 0.108 µg/L. Three samples were over the permission limit (0.05 µg/L) (15). Zinedine, A. *et al*, Jordi investigated 54 samples of pasteurized milk produced in 5 different dairies from Morocco for the presence of AFM_1_ using immunoaffinity columns, liquid chromatography, fluorescence. Their results showed that 88.8% samples were contaminated with AFM_1_; 7.4% were above the maximum level of 0.05 µg/L set by Moroccan and European regulations for AFM_1_ in liquid milk. The incidence of AFM_1_ in milk from these 5 different dairies were 100, 92.3, 90, 83.3, and 77.7% respectively, with AFM_1_ levels ranging from 0.001 to 0.117 µg/L and a mean value of 0.0186 µg/L (16).

Tekinsen, K. Kaan and Eken, H. Semih analyzed 100 UHT milk and 132 Kashar cheese samples from retail outlets in 5 large cities (Istanbul, Izmir, Konya, Tekirdag, and Edirne) for AFM_1_ by using ELISA. Sixty-seven percent UHT milk samples and 82.6% Kashar cheese samples contained AFM_1_. The incidence of AFM_1_ in the UHT milk and Kashar cheese samples ranged from 0.010 to 0.630 µg/kg and from 0.050 to 0.690 µg/kg, respectively. AFM_1_ levels in 31 (31%) UHT milk samples and 36 (27.3%) Kashar cheese samples exceeded the maximum tolerable limit proposed by EC and TFC. AFM_1_ levels in the samples indicate high aflatoxin levels, thereby constituting a human health risk in Turkey (17). Srivastava, V. P *et al*. measured 54 samples of fresh full cream and skimmed skim milk, powdered milk, yogurt, and infant formula for AFM_1_ by using HPLC after sample clean-up using immune affinity columns in Kuwait. A total of 28% of samples were contaminated with AFM_1_, with 6% above the maximum permissible limit of 0.2 µg/L. According to their results, 3 fresh cow milk samples collected from a private local producer showed the highest level of 0.21 µg/L AFM_1_. There was no contamination with AFM_1_ in powdered milk and infant formula (18).

In 1984, Piva, G. *et al*. tested 313 samples of imported liquid milk and 159 samples of imported cheese for AFM_1_; 225 milk samples were obtained from Federal Republic of (FR) Germany and 88 from France, while 82 cheese samples were obtained from France, 34 from FR Germany, and 43 from the Netherlands. The number of positive samples was low for both German (13.8%) and for French (12.5%) milk, and the contamination levels were very low (maximum 23 ng/L). As regards the cheeses, AFM_1_ was detected in 19.5, 26.5, and 53.5% French, German, and Dutch samples, respectively, but only 2 French samples exceeded 250 ng/kg (the limit set by Swiss law). In 1985, 2 surveys were carried out on 276 milk samples mostly obtained from individual farms and on 416 cheese samples obtained from all parts of the country. As regards the milk samples, 70 (25.3%) contained AFM_1_, but generally at very low levels; in fact only 7 (2.5%) samples exceeded 50 ng/L. AFM_1_ was found in 130 (31.3%) cheese samples, but again only 9 (2.2%) exceeded 250 ng/kg. There was no significant difference in AFM_1_ levels between Italian, German, and French cheese samples, but these were significantly lower (*P* < 0.01) than in Dutch samples (19).

Sefidgar, S. A. *et al*. collected raw cow’s milk samples from milk churns at 40 traditional and semi-industrial cattle farms located in Babol (Northern Iran) in the winter of 2006. In total, they analyzed 120 raw milk samples for AFM_1_ contamination by ELISA. Sixty-eight out of 120 samples (56.7%) had AFM_1_ levels ranging from 50 to 352.3 ng/L. Fifty-two samples (43.3%) contained AFM_1_ at 4–50 ng/L. AFM_1_ contamination levels were 4–352.3 ng/L with an average of 102.73 ng/L. Their results indicated that 56.7% of samples were above the limit of European community regulations (0.050 µg/L). In other words, AFM_1_ contamination levels in raw milk were more than twice as high as permitted levels (20).

Mohamadi Sani, A. *et al*. evaluated AFM_1_ contamination and antibiotic presence in milk samples in the Khorasan province in Iran. For 4 months (March to June 2008), 196 milk samples were collected from 7 dairies. The presence and concentration range of AFM_1_ in the samples were investigated by ELISA. AFM_1_ was found in 100% of the examined milk samples with an average concentration of 0.07792 µg/kg. The concentrations of AFM_1_ in all samples were lower than the Iranian national standard and the FDA limit (0.5 µg/L), but 80.6% samples had an AFM_1_ level greater than the maximum limit (0.050 µg/L) accepted by the European Union and the Codex Alimentarius Commission. There was no significant difference between the mean AFM_1_ concentrations in the milk samples obtained from different factories (*P* > 0.05) (21).

Heshmati, Ali *et al*. determined the levels of AFM_1_ in 210 UHT milk samples obtained from supermarkets in Tehran, Iran by using ELISA. AFM_1_ was found in 116 (55.2%) of 210 UHT milk samples. The levels of AFM_1_ in 70 (33.3%) samples were higher than the maximum limit (0.05 µg/L) accepted by Iran and some European countries, while none of the samples exceeded the prescribed limit of US regulations. The highest mean concentration of AFM_1_ was recorded at 0.087 µg/L and the lowest at 0.021 µg/L. The incidence of AFM_1_ levels exceeding legal limits in UHT milk samples (33.3%) was much higher relative to some other countries. It was therefore concluded that the levels of AFM_1_ in the UHT milk samples in Iran were high and seemed to pose a threat to public health (22).

The results of this study showed that all 100 investigated pasteurized milk samples were contaminated with AFM_1_ at levels ranging from 0.45 to 9.7 ng/L (mean, 2.7 ng/L). Therefore, all milk samples contained AFM_1_ below the maximum limit of 50 ng/L for milk in Iran. These results highlight the necessity of a survey involving a larger number of milk and milk product samples, and suggest that currently, the contamination of milk and milk products with AFM_1_ does not appear to pose a serious health problem to Ahvaz city in the Khozestan province of Iran. Nevertheless, a continuous surveillance program may be warranted to monitor the occurrence of aflatoxins in animal feeds responsible for the present limited contamination. In addition, prolonged storage of cereal and nuts in warm and humid conditions should be avoided in order to minimize the risk of aflatoxin contamination.
